# Post-Earthquake Building Evaluation Using UAVs: A BIM-Based Digital Twin Framework

**DOI:** 10.3390/s22030873

**Published:** 2022-01-24

**Authors:** Nathaniel M. Levine, Billie F. Spencer

**Affiliations:** Department of Civil and Environmental Engineering, University of Illinois at Urbana-Champaign, Urbana, IL 61801, USA; nlevine3@illinois.edu

**Keywords:** unmanned aerial vehicles, building information modeling, digital twin, computer vision, post-earthquake evaluation, automated inspection

## Abstract

Computer vision has shown potential for assisting post-earthquake inspection of buildings through automatic damage detection in images. However, assessing the safety of an earthquake-damaged building requires considering this damage in the context of its global impact on the structural system. Thus, an inspection must consider the expected damage progression of the associated component and the component’s contribution to structural system performance. To address this issue, a digital twin framework is proposed for post-earthquake building evaluation that integrates unmanned aerial vehicle (UAV) imagery, component identification, and damage evaluation using a Building Information Model (BIM) as a reference platform. The BIM guides selection of optimal sets of images for each building component. Then, if damage is identified, each image pixel is assigned to a specific BIM component, using a GrabCut-based segmentation method. In addition, 3D point cloud change detection is employed to identify nonstructural damage and associate that damage with specific BIM components. Two example applications are presented. The first develops a digital twin for an existing reinforced concrete moment frame building and demonstrates BIM-guided image selection and component identification. The second uses a synthetic graphics environment to demonstrate 3D point cloud change detection for identifying damaged nonstructural masonry walls. In both examples, observed damage is tied to BIM components, enabling damage to be considered in the context of each component’s known design and expected earthquake performance. The goal of this framework is to combine component-wise damage estimates with a pre-earthquake structural analysis of the building to predict a building’s post-earthquake safety based on an external UAV survey.

## 1. Introduction

In the aftermath of large earthquakes, buildings in the affected region must be evaluated for structural integrity and other life safety hazards before occupants can safely return to their homes and places of work. Typically, inspectors survey a building for damage indicating degradation to the vertical or lateral load carrying systems, as well as for nonstructural hazards. In the United States, the procedures for post-earthquake building inspection are described in ATC-20 [1,2]. After the inspection, earthquake-affected buildings are assigned placards classifying them into three categories: (1) inspected (green), no apparent hazards or loss of load carrying capacity; (2) restricted use (yellow), building specific restrictions are indicated on the placard and are to be enforced by the owner; and (3) unsafe (red), extreme life safety hazard or imminent collapse danger, no entry permitted. This effort requires a team of experienced inspectors, comprised of structural engineers and building officials, to classify the safety of every building in the affected region.

The first step in the inspection process is a rapid evaluation of the building exterior. A team of inspectors classifies the building based on obvious signs such as partial collapse, severe leaning, damaged structural members, damaged parapets and chimneys, or chemical and electrical hazards. If a building does not clearly meet the criteria for a green or red tag, it is assigned a yellow tag. A yellow-tagged building undergoes a subsequent detailed evaluation of the building’s interior, which requires identification and assessment of the vertical and lateral load carrying systems and inspection for overall condition, foundation damage, residual drift, and hazardous materials [1,2]. However, inspectors are not permitted to perform any destructive investigations, such as removing architectural finishes, without owner consent [1], leaving inspectors to infer structural condition from nonstructural damage, based on their own experience and judgment. The problem is compounded by a lack of a centralized database of documentation for the buildings being evaluated [3]. If the building’s condition is still uncertain after the detailed inspection, a subsequent engineering evaluation, requiring analysis by a structural engineer, is performed.

Manual post-earthquake evaluation can be limited by the need to safely mobilize teams of qualified inspectors. The emergency demands can overwhelm local jurisdictions, requiring the organization and deployment of non-local inspection teams, which can take weeks [4]. Moreover, field inspectors can face unsafe conditions in a post-earthquake environment, ranging from falling and collapse hazards to chemical and electrical dangers. Emergency search and rescue resources, therefore, must be devoted to ensuring inspector safety in addition to other emergency response duties [3]. These examples highlight three major limitations of the current approach for post-earthquake evaluation of buildings: (1) subjectivity, (2) speed, and (3) safety.

In response to these limitations for post-earthquake inspection, some jurisdictions allow structural engineers to develop building assessment plans in advance of an earthquake. In the event of an earthquake, the engineer performs an inspection within hours, rather than days or weeks, with the goal of reducing post-earthquake downtime for businesses and allowing buildings to be reoccupied more quickly. For example, San Francisco’s Building Occupancy Resumption Program [5] allows owners to hire an engineer to develop a post-earthquake inspection plan, which the city reviews and certifies. Recognizing the benefit of inspectors with direct knowledge of the specific building design, other cities in California, including Berkeley and Glendale, have also introduced similar programs [6,7]. However, under these programs, a person needs to physically visit a building and therefore they suffer from the same limitations as traditional manual inspection.

To reduce reliance on manual inspection, researchers have developed methodologies for automated post-earthquake building inspection using unmanned aerial vehicles (UAVs) and computer vision [8,9]. Such systems use UAVs to rapidly collect exterior images of a target building. Computer vision techniques then automatically identify damage on the building exterior. Such algorithms use image processing pipelines to identify damage such as concrete cracking, spalling, and exposed rebar [10,11,12,13,14]. More recently, classifiers using convolutional neural networks [15,16,17,18,19,20] have successfully been applied to automatically identify structural component type and structural damage. Beyond identifying structural damage, Paal et al. [14] developed a classifier to automatically estimate maximum column drift demand experienced during an earthquake from an image of a damaged column. However, to make meaningful decisions about the overall safety of the building requires additional information about the role of the damaged components within the whole structural system. To fully automate the inspection process, damage identified in images must be localized to specific building components.

Tying damage to specific components enables that damage to be considered in the context of the components’ design, connectivity, and function and thus allow the overall performance of the building to be assessed. Methods have proposed using principles from performance-based earthquake engineering to predict post-earthquake building safety. For example, Mitrani-Reiser et al. [21] estimated the probability of an earthquake-affected building receiving an unsafe placard based on the measured earthquake intensity. In addition, statistical and machine learning-based classifiers have been developed to predict a damaged building’s relative reduction in collapse capacity based on simulated damage using component fragility curves and seismic demands on the structure [22,23,24,25]. However, these methods are intended for risk assessment and for calibrating building tagging criteria to meet specific performance targets, rather than for assessing the performance of an individual building. Future efforts to incorporate UAV observations from the building exterior into such a classifier will require observed damage to be associated with specific building components. As noted previously, current computer vision-based damage assessment methods do not provide context for the observed damage. This paper, therefore, develops a framework to automatically associate damage identified from UAV surveys with specific building components to enable assessment of the global safety of the building.

Building Information Models (BIMs), which store both geometric and semantic data about a building, offer the potential to provide contextual information for UAV surveys. Researchers have combined BIM with as-built photographic surveys to monitor construction progress [26,27,28,29]. Such approaches typically generate a 3D reconstruction from site photographs aligned in the BIM geometric reference frame. Once aligned, semantic information stored in the BIM is used to track construction progress. For example, the user can assess, on an element-wise basis, whether construction is on schedule and whether the as-built geometry conforms to the design. BIM can fill a similar role for post-earthquake inspection, providing a way to store data, establish a geometric reference frame for UAV imagery, and efficiently organize and classify structural components by overlaying BIM on photographs. Using BIM requires either a modern target building, where a BIM is created as part of the design process, or sufficient stakeholder investment to justify creating a BIM to aid in post-earthquake inspection. On its own, however, BIM is not predictive; to predict global damage and future performance, the BIM and visual survey must be combined with an analytical model of the building.

Digital twins have been proposed as a means to provide predictive information about an as-built structure. As used herein, a digital twin is a physics-based probabilistic simulation model that is continuously updated using sensor information and load history from the physical system [30]. Such systems have been proposed and deployed for manufacturing, industrial, aerospace, and civil engineering applications [31,32]. In civil structural health monitoring, frameworks have been proposed that meet the criteria of a digital twin. For example, Hughes et al. [33] described a probabilistic system for risk assessment and decision making using sensor data and demonstrate its application on a laboratory truss structure. Zhu et al. [34] developed a real-time digital twin framework for structural health monitoring applications using vibration data, whereby the digital model can be continuously and efficiently updated to reflect changes to the structure. Gardner et al. [35] proposed a scheme to characterize and predict unanticipated nonlinear acceleration responses with a digital twin. However, while useful for updating a digital twin for predicting seismic response, many of these examples have been limited to simulation or scaled laboratory experiments. Angjeliu et al. [36] developed a digital twin model of a historic cathedral in Milan, Italy, and successfully predict existing damage using their calibrated digital twin to model the evolution of the structure through its lifetime. Such a model has the potential for predicting future performance under extreme loads and directing repair and maintenance operations. Lin et al. [37] developed a digital twin of a scale model of the Sutong Bridge in China, specifically for characterizing seismic performance. By testing a physical scale model to failure under increasing earthquake excitations, the authors demonstrated finite element model updating procedures to accurately predict the failure modes and intensity measures of the physical specimen using the digital twin. However, while successful at predicting collapse or existing damage to the structure, neither [36] nor [37] employed the digital twin to incorporate observed damage and revise collapse predictions for the earthquake-damaged structure. Indeed, digital twins have yet to be fully exploited for the post-earthquake assessment problem.

This paper proposes a BIM-based digital twin framework to tie computer vision-derived damage observations to specific structural and nonstructural components of the target building. BIM plays an integral role in this framework: it will serve as a reference frame for registering UAV photographs and 3D point cloud reconstructions, enabling damage identified on the building’s exterior to be tied to specific structural and nonstructural building components with predetermined models for expected damage progression as a function of seismic demand. Combined with a pre-earthquake structural analysis to predict individual components’ relative contributions to global building strength and stability, the digital twin will ultimately enable rapid decision making in the post-earthquake environment. First, an overview of the proposed digital twin framework is presented. The methodology for connecting the BIM and UAV survey to guide the building assessment is discussed. To demonstrate the proposed approach, an example will be presented of a reinforced concrete moment frame building on the University of Illinois campus in Urbana, Illinois. A second example, using a synthetic graphics environment to impose damage on a building, will demonstrate how the digital twin is used for BIM-guided 3D change detection for assessing nonstructural damage. Finally, a discussion is provided describing how the proposed BIM-based digital twin will be integrated with a structural analysis to predict the reduced capacity of the earthquake-damaged building. The framework presented will ultimately allow for automated decisions regarding a damaged building’s based on images acquired from a UAV.

## 2. Methodology

**Figure 1:**
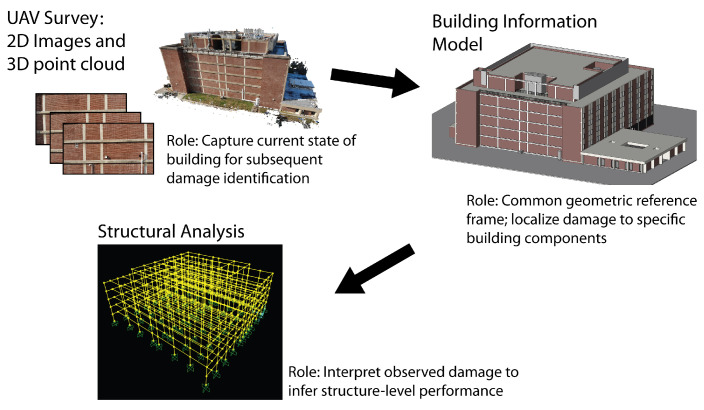
Schematic overview of the components of the digital twin system.

This section describes the steps to develop the proposed digital twin framework. An overview of the digital twin components is presented in Figure 1. The first step in constructing the digital twin occurs before the earthquake with an initial assessment of the building, including a walkthrough, drawing review, and structural analysis. A BIM of the as-built building shell is then developed using commercially available software. Subsequently, an initial photographic survey of the building is flown and a 3D point cloud of the target building is created and aligned with the BIM. This combination of aligned BIM and point cloud, set of survey images, and structural analysis model, forms the digital twin. After an earthquake occurs, a second, post-earthquake UAV survey is conducted, and an updated 3D point cloud is generated from the survey images. The digital twin guides selection of an optimal set of images from the survey and identifies building components in the images. The digital twin also applies 3D change detection between the pre- and post-earthquake point clouds to identify damaged nonstructural components. By tying damage to individual building components, each component can be considered in terms of its individual expected performance to infer component-level demands and subsequently building-level performance and safety. The following sections describe each step of this process in detail.

### 2.1. Pre-Earthquake

Before an earthquake occurs, the baseline digital twin is assembled. At this stage, as shown in Figure 1, the digital twin consists of a set of UAV photos, a 3D point cloud generated from those photos, a BIM, and a structural analysis model. The procedure begins with a preliminary assessment of the building to inform BIM and analysis model development, as well as an initial UAV survey to capture the baseline state of the building.

#### 2.1.1. Preliminary Building Assessment

The initial assessment investigates the building design, as-built state, and expected earthquake performance. The engineer first reviews the design drawings and visits the building to identify both structural and nonstructural components on the building exterior that may be damaged during an earthquake and would be visible in a UAV survey. A structural analysis is performed to further identify structural damage-sensitive components and identify potential structural collapse mechanisms. In this context, damage-sensitive components are structural and nonstructural building components that may be damaged during an earthquake, are visible from the exterior of the building, and may be indicative of damage on the building interior. While the exact nature of the analysis will vary depending on construction type, a performance-based framework, such as described in FEMA P58 [38], should be used to identify structural components that are expected to be damaged and how those individual components affect global structural strength and stability. For each damage-sensitive component, both structural and nonstructural, an appropriate component fragility curve, or family of curves, is selected or computed. The component fragility curves define the probability of meeting or exceeding a discrete, qualitative damage state and will enable any observed damage to be linked back to the structural analysis model after an earthquake occurs.

#### 2.1.2. BIM Development

Next, a BIM is developed for the digital twin in which the damage-sensitive components are explicitly modeled. While the geometry should be modeled as accurately as possible, details such as bolted connections or individual rebar need not be represented. Structural elements should approximately conform to BIM Level of Detail (LOD) 300 [39], where the shape and location are modeled precisely. Nonstructural elements should conform to LOD 200 at minimum, where the geometry is modeled approximately. Typically, a model developed during the building design process is sufficient, provided that exterior damage-sensitive components are modeled. For any exterior structural components modeled in the BIM, the corresponding elements in the structural analysis model should be identified. Typically, in the proposed application, the BIM is developed using commercial software such as Autodesk Revit [40], and exported to the open Industry Foundation Classes format [41] for incorporation into the digital twin.

#### 2.1.3. UAV Survey and 3D Reconstruction

A photographic UAV survey of the target building is flown, and the collected images are used to create a 3D point cloud of the building, termed herein as the 3D reconstruction. The survey is planned so that the damage-sensitive components identified in the preliminary assessment will be imaged. The building should be photographed from a close enough distance that the smallest damage to be considered can be resolved. Following the survey, the images are input to a 3D reconstruction pipeline, including structure from motion for sparse reconstruction [42] and multi-view stereo for dense reconstruction [43]. Only those images that are successfully registered in the 3D reconstruction are used in the subsequent methodology. This pipeline calibrates the UAV images, computing a set of intrinsic and extrinsic camera parameters for each image. The extrinsic parameters locate the camera in the 3D point cloud reference frame. By transforming the point cloud to the BIM reference frame, the location and orientation of each image can be defined in the BIM reference frame. The transformation between the point cloud and BIM is determined by selecting manual point correspondences between the two models. This transformation, represented by a 4 × 4 transformation matrix, includes a rotation, translation, and scaling. The collection of BIM, transformed point cloud, UAV images, structural analysis model, and individual component performance models form the baseline digital twin.

### 2.2. Post-Earthquake

Following an earthquake, a UAV survey is again flown to capture a set of images of the earthquake-affected building. A post-earthquake point cloud is generated from the images and manually registered with the BIM using the same procedure as the pre-earthquake point cloud. The transformation matrix, **T**, is computed as part of the registration and is used in the subsequent steps. The new set of images and point cloud is included as part of the digital twin. The BIM is used to guide image selection for subsequent damage identification and to associate any damage with specific damage-sensitive components.

#### 2.2.1. BIM-Guided Automatic Image Selection by Component

The first step in this process is to use the BIM to automatically select images of the damage-sensitive building components from the hundreds or thousands of UAV images collected during the post-earthquake survey. The objective here is to determine a set of images of a given building component that ensures maximal coverage for subsequent damage assessment.

The automatic image selection methodology is based on previous work in UAV path planning. One approach to UAV path planning is to use the BIM model as an a priori representation of building geometry to plan and evaluate proposed paths for the UAV surveys [44,45,46]. Ibrahim et al. [45] assigned each face of each building component a unique (R, G, B) color identifier and simulated a UAV flight. Then, the proposed path was evaluated based on whether a component is adequately covered by the acquired imagery by counting the number of visible pixels of that component’s unique color. This metric is applied in this paper to evaluate the prominence of a building component in a UAV image.

**Figure 2:**
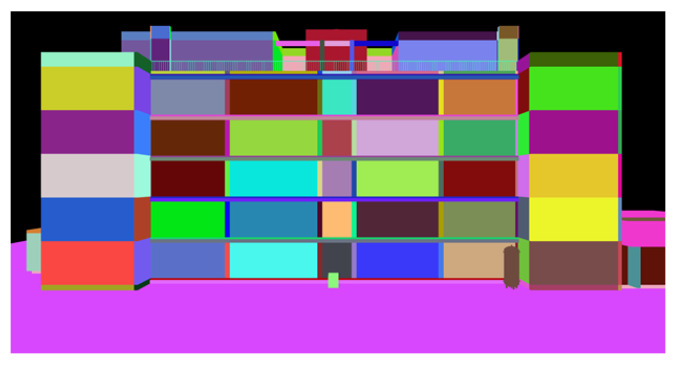
Elevation view of a BIM. Each component has a unique RGB color identifier.

Figure 2 shows the elevation of the rendered BIM for a typical building, where each BIM component is assigned a unique color identifier. First, the BIM file is parsed and its geometry converted to a triangle mesh using IfcOpenShell in Python [47]. The elements in the mesh corresponding to each BIM component are assigned a unique (R, G, B) color identifier as well as one of three classes, (1) structure (beams and columns), (2) nonstructural walls, and (3) mechanical, electrical and plumbing (MEP). The color-coded mesh is displayed in Python using Open3D [48]; the BIM can be rendered from the perspective of any UAV image used in the 3D point cloud reconstruction. A minimum of five canonical exterior views of the building are established: four elevations in each cardinal direction and one plan view of the roof, that show the full extent of the building. The camera matrix for each of these canonical views, $P_{\mathrm{can}}$, is defined by the user.

The 3D reconstruction pipeline outputs a list of images used in the point cloud reconstruction, each with a camera matrix **P** parametrized by the intrinsic camera matrix **K**, and extrinsic matrix, defined by a rotation matrix, **R** and a translation vector, **t** [42]:

(1) P=K[R|t]

The extrinsic parameters are defined relative to the reconstruction reference frame. The point cloud in the reconstruction reference frame is transformed to the BIM reference frame by a 4 × 4 transformation matrix **T**. The extrinsic camera matrix is converted to the BIM reference frame by the relation:

(2) $$\left[R_{\mathrm{BIM}}|t_{\mathrm{BIM}}\right]=\left[R|t\right]T^{-1}$$

where $R_{\mathrm{BIM}}$ and $t_{\mathrm{BIM}}$ are the camera’s rotation matrix and translation vector in the BIM reference frame. For a given input image with known K, $R_{\mathrm{BIM}}$ and $t_{\mathrm{BIM}}$, the color-coded BIM can be rendered from the same perspective in the Open3D visualizer. Following [45], the number of pixels of each unique color is counted in the rendered BIM to create a list of imaged components, based on their unique RGB identifier. This count determines how prevalent a component is in the image. Only elements with a minimum count of 500 pixels are considered to ensure the component of interest is sufficiently visible in the image. By iterating through all images used in the reconstruction, a dictionary is created that maps from an image to a list of components contained in the image. To select images, this dictionary is inverted to map from unique building component to a list of images where that component appears.

**Figure 3:**
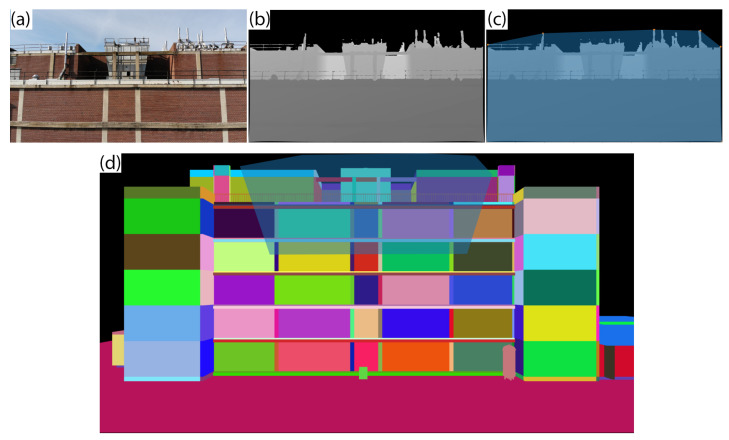
Example UAV image (**a**), depth map (**b**), and depth map with convex hull highlighted (**c**). The vertices of the convex hull are projected on to the canonical building elevation (**d**).

For each damage-sensitive component, a set of images {$I_{1}$, …, $I_{n}$} is determined. For a component *C*, all images containing *C* are ranked in order of number of pixels that correspond to *C*, based on the RGB identifier. The number of pixels is a proxy for how prominent the component is in an image. The first image, $I_{1}$, is the image with the most pixels of *C*. The extent of $I_{1}$ is projected on to the appropriate canonical building view, $I_{C}$. To project the image, a corresponding depth image, $D_{1}$ is used, as shown in Figure 3. $D_{1}$ is automatically output as part of the 3D reconstruction process. The usable extent of $D_{1}$ is determined by the convex hull of the nonzero points, as zero (black) values indicate infinite distance. The 3D locations of the convex hull vertices in the camera reference frame, $X_{\mathrm{cam}}=\left(X_{cam},Y_{cam},Z_{cam}\right)$ are then estimated by the relation [48]:

(3) $$\begin{array}{c} Z_{cam}=d \end{array}$$

(4) $$\begin{array}{c} \;\;\;\;\;\;\;\;\;\;\;\;\;\;\;\;\;\;\;\;\;\;\;\;\;\;\;X_{cam}=\left(u-c_{x}\right)*Z_{cam}/f \end{array}$$

(5) $$\begin{array}{c} \;\;\;\;\;\;\;\;\;\;\;\;\;\;\;\;\;\;\;\;\;\;\;\;\;\;\;Y_{cam}=\left(v-cy\right)*Z_{cam}/f \end{array}$$

where (u,v) is the 2D image coordinate of the vertex in the input image, *d* is the value of the depth image at the vertex coordinate, and $c_{x}$, $c_{y}$, and *f* are the principal point coordinates, and focal length in pixels, which are determined from the camera intrinsic matrix [42]. Then, the 3D coordinates each vertex in the BIM reference frame, $X_{\mathrm{BIM}}$, are computed and projected to the 2D point $u_{\mathrm{can}}$ on the canonical building elevation:

(6) $$\begin{array}{c} \;\;\;\;\;\;\;\;\;\;\;\;\;\;\;\;\;\;\;\;X_{\mathrm{BIM}}=R_{\mathrm{BIM}}^{T}\left(X_{\mathrm{cam}}-t_{\mathrm{BIM}}\right) \end{array}$$

(7) $$\begin{array}{c} u_{\mathrm{can}}=P_{\mathrm{can}}X_{\mathrm{BIM}} \end{array}$$

The bottom image in Figure 3 shows an example of the UAV image extent projected onto the color-coded building elevation. First, image $I_{1}$ is projected onto the color-coded BIM elevation. The number of pixels of element *C* covered by the projection of $I_{1}$ is compared to the total number of pixels. Next, the image with the second highest number of pixels containing component *C*, $I_{2}$, is projected, continuing until all pixels of *C* are covered by the image projections, or the list of images containing *C* is exhausted.

The result is a set of optimal images for each damage-sensitive component on the building’s exterior, which can be used for subsequent damage assessment. For example, the images can be input to an image processing pipeline for damage identification, like [14], or to a neural network for damage detection, like [9,18]. The specific damage detection methodology is outside the scope of this paper. Damage detection should take advantage of pre- and post-earthquake building surveys to identify new changes associated with the earthquake, using a change detection method like [49]. For example, the system should be able to distinguish between previously exposed rebar resulting from environmental degradation and newly exposed rebar from heavy earthquake damage. Identified damage then needs to be linked to a building component to interpret the impact on building performance.

#### 2.2.2. BIM-Guided Component Identification in 2D Images

Subsequently, damage identified from the images is linked to a building component. Previous studies [15] have demonstrated using CNNs for structural component type identification. However, to link damage with individual elements in the BIM, and eventually, the structural analysis model, the component type and unique identifier must be determined. The same procedure described in the previous section is used to render 2D views of the BIM model from the same perspective as the UAV images. This color-coded BIM image can be used as a prior label estimate for each pixel in the target image. However, registration errors between the BIM and point cloud and geometric modeling errors in the BIM can cause misalignment between the image and the rendered BIM perspective. Therefore, a Markov random field-based segmentation scheme using GrabCut [50] is used to refine the initial component labels from the BIM.

GrabCut [50] is a foreground segmentation algorithm that relies on limited user interaction to separate foreground and background pixels using graph cuts. The user specifies initial background and foreground pixels, typically by drawing a box around the foreground object. With these initial assignments, GrabCut estimates a probability distribution of RGB values for foreground and background. Pixel labels are assigned iteratively based on an energy minimization, where the total energy is a sum of unary and pairwise potentials. The unary potential indicates how likely a pixel belongs to a certain class; the pairwise potential encourages coherence in groups of neighboring pixels of similar RGB intensities. An optimal set of foreground/background labels is estimated with a minimum cut algorithm [51]. The algorithm continues iteratively, alternating between estimating a new probability distribution for the foreground and background pixels, and then assigning optimal pixel labels. For this study, OpenCV’s [52] GrabCut implementation is used. Rather than the user drawing a box, the rendered BIM perspective serves as an initial foreground label estimate.

**Figure 4:**
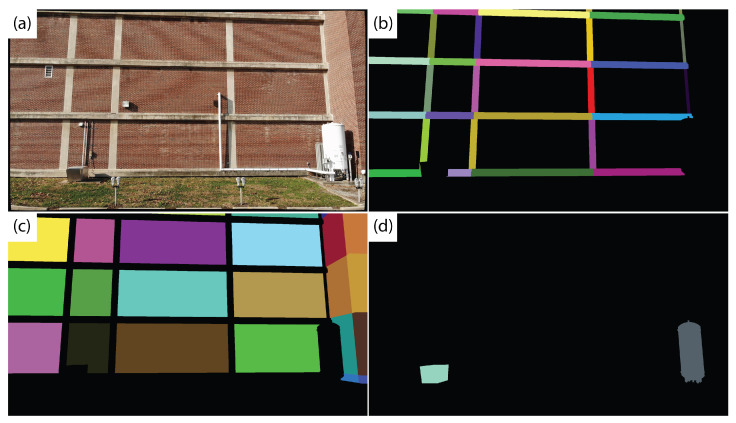
Original input image from the UAV survey (**a**), with the structure (**b**), wall (**c**), and MEP (**d**) masks generated from the BIM.

For the segmentation, three classes of building elements are considered: structural, MEP, and wall/background. Each object type in the BIM is assigned a corresponding class. For each class, the colors of all non-class objects are set to black to create a mask corresponding to that class. Example masks for the structural, MEP and wall classes are shown in Figure 4.

**Figure 5:**
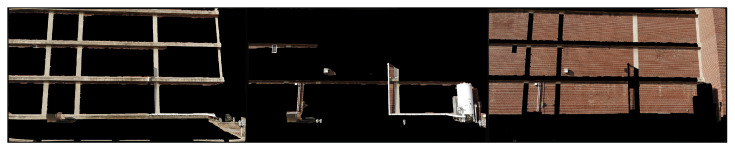
Intermediate masks output for each category by the GrabCut algorithm. From left to right: structure, MEP, walls.

However, the alignment between the UAV image and rendered BIM perspective is imperfect. Therefore, using these masks as initial foreground labels, GrabCut is run to refine each segmentation mask. Figure 5 shows the refined GrabCut segmentation masks. Note that overlap occurs between the masks due to the initial misalignment and because GrabCut groups pixels based on color intensity. For example, the initial structural element mask includes portions of the brick wall, in particular areas in shadow. Therefore, the GrabCut algorithm assigns shadowed regions of the wall to the foreground class in the structure mask (Figure 5).

**Figure 6:**
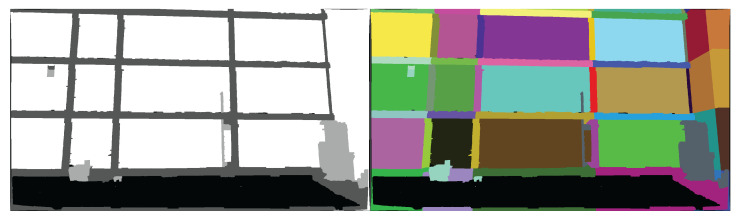
Sample output class (**left**) and element label (**right**) maps from the BIM-guided element identification method.

Each foreground pixel in each of the structural, MEP, and wall masks is assigned to a particular BIM element using a nearest neighbor classifier. The basis for labels are the initial BIM masks (Figure 4). Each of the three classes is considered separately, such that if a pixel is labeled as foreground in multiple class masks, it will initially be assigned multiple labels. For a specific foreground pixel for a particular class, the assigned label is the label of the nearest (in pixel coordinates) labeled pixel in the corresponding BIM mask. The distance from the foreground pixel to the nearest BIM mask pixel is recorded. Each foreground pixel in each of the three class maps is assigned a label and corresponding distance. Then, if a pixel has multiple assigned classes, the class and element label with the minimum distance is assigned to that pixel to produce a class label map (Figure 6, left) and element label map (Figure 6, right). To account for components segmented by GrabCut but not modeled in the BIM, a maximum distance threshold is assigned, beyond which any pixel is assigned as background. Any pixels with identified damage will then be associated with a specific BIM element for subsequent building condition assessment.

#### 2.2.3. Damage Detection in 3D Point Clouds

Certain types of nonstructural damage, such as façade collapse and MEP equipment sliding and overturning, are associated with large displacements that can be detected as changes in the pre- and post-earthquake 3D point clouds. Point cloud change detection has previously been used to infer damage on structures. For example, Ghahremani et al. [53] generated dense 3D point clouds of structural components before and after damage. Direct comparisons of the point clouds successfully localized and estimate damage severity. The authors also demonstrated finite element updating through dense point cloud comparison. Khaloo et al. [54] demonstrated a similar methodology on a large gravity dam, conducting a UAV survey and creating a 3D point cloud before and after applying artificial damage to the structure. Both of these studies quantified volumetric changes to the structure using direct comparison between the pre- and post-damage point clouds. Rather than directly quantify the volumetric changes to a structure, this study identifies large volumetric changes due to nonstructural damage, such as a wall collapse or mechanic unit overturning, and assigns a binary damaged/not damaged label to specific nonstructural building components. This type of damage is typically caused by high floor accelerations or large interstory drifts, and assigning a component level will be used to infer maximum seismic demands on the structure. Changes are detected by component, based on its corresponding geometry in the BIM.

In the proposed approach, several preprocessing steps are applied before comparing the two point clouds. First, both point clouds are downsampled to a uniform voxel grid. Then, each point cloud is cropped to match the extent of the other. Statistical outlier removal is performed [48] to remove spurious points. After the initial preprocessing, a component-wise assessment is performed to determine the state of each nonstructural external building element.

The BIM geometry is used to direct the nonstructural assessment. For a particular building component, each point cloud is cropped to the extent of the BIM element’s bounding box. The dimensions of the bounding box are increased by 10–20% to account for misalignment between the BIM and point clouds. The increase in bounding box size is selected based on component type. Cropping the clouds to the extent of each element helps avoid detecting spurious changes in poorly reconstructed regions, such as the edges of the cloud and around fine details. The distance between the two point clouds is computed using Open3D. For change detection, one cloud is designates as the source cloud and the other is designated as the target. For a given point in the source point cloud, the distance to the target cloud is defined as the Euclidean distance to the nearest point in the target cloud. Statistics of the distance measures for all points within the cropped region are considered to determine the state of the component. For example, damaged components will tend to have a higher mean or standard deviation of distance relative to undamaged components. Each nonstructural component can then be assigned a binary damaged/not damaged label based on a predetermined threshold.

The BIM, either through 2D component identification or 3D change detection, enables the digital twin to associate any observed damage with specific building components. The result of the application of these methods will be a list of damage-sensitive components and qualitative descriptions of damage that can be considered to assess the global safety and performance of the target building. While the full methodology cannot be demonstrated from start to finish without pre- and post-earthquake surveys of a target building, the following examples demonstrate the BIM-guided image selection, BIM-guided component identification, and 3D change detection methods of the digital twin system.

## 3. Example 1: BIM-Based Digital Twin Development for a Reinforced Concrete Moment Frame Building

This example illustrates the development of the BIM-based digital twin and demonstrates BIM-guided automatic image selection and component identification using an existing reinforced concrete moment frame building, Turner Hall, located in Urbana, Illinois. First, a description of the building is presented. To identify damage-sensitive nonstructural components on the building exterior, an initial assessment is performed based on a drawing review and building walkthrough. Subsequently, a nonlinear structural analysis model is developed to identify damage-sensitive structural components. Based on the assessment, a BIM is developed containing the identified damage-sensitive components. A UAV survey is flown and input to a 3D reconstruction pipeline to generate a 3D point cloud of the building. The BIM and 3D reconstruction are used to demonstrate the BIM-guided automatic image selection and BIM-guided component segmentation described in Section 2. Each of these steps is described in detail below, followed by results of the two methods applied to representative building components.

### 3.1. Building Description

**Figure 7:**
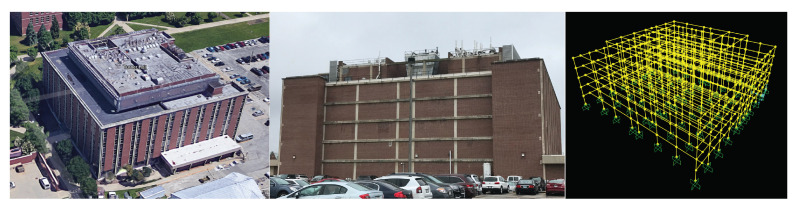
(**Left**): Aerial view of Turner Hall from Google Maps [55]. (**Center**): East elevation of Turner Hall showing the exposed reinforced concrete moment frame. (**Right**): Structural analysis model of Turner Hall showing the entire moment frame system.

Turner Hall houses the Department of Crop Sciences at the University of Illinois at Urbana-Champaign. The building was initially constructed in 1961 and expanded in 1975. The structural system is a nonductile concrete moment frame that serves as both the vertical and lateral force resisting systems, with one-way flat slab concrete floors. The building has five stories, in addition to a basement and mechanical penthouse. The moment frame is exposed on the east elevation of the building (Figure 7).

### 3.2. Preliminary Building Assessment

**Figure 8:**
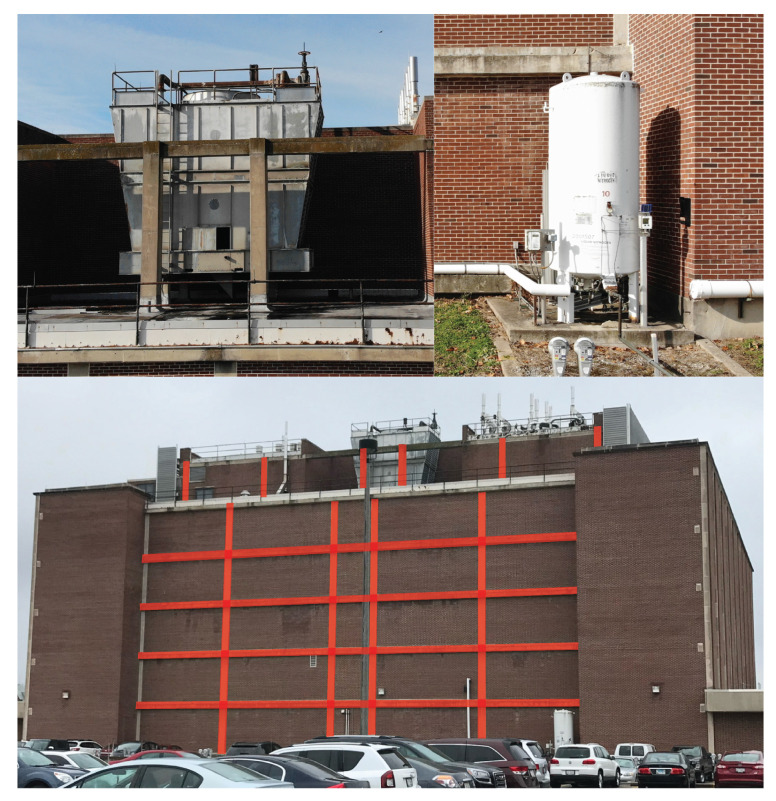
Damage-sensitive components. The top images show acceleration-sensitive mechanical equipment at the roof (**left**) and ground levels (**right**). The bottom image shows the building elevation with damage-sensitive beams and columns highlighted.

The initial assessment of Turner Hall is performed, including a drawing review, building walkthrough, and structural analysis. Damage-sensitive nonstructural components were identified, including several mechanical units on the building exterior (e.g., the rooftop cooling tower and ground-level storage tank), as shown in Figure 8. Both units are considered as acceleration-sensitive components. Additionally, each individual brick façade panel is considered as a damage-sensitive component, subject to damage by excessive interstory drift when motion is parallel to the wall and subject to damage by high accelerations when motion is perpendicular to the wall.

A nonlinear structural analysis model is developed to identify structural damage-sensitive components that can be observed from external imagery. Nonlinearity is accounted for using concentrated plastic hinges at the ends of beams and columns, with cyclic backbone curves as defined by ASCE 41-13 [56]. A total of 42 ground motions are selected from the FEMA P695 [57] far field record set for nonlinear time history analysis. Each record was scaled to 0.5, 1.0, 1.5, 2.0, 2.5, 3.0, 3.5, and 4.0 times the base record, for a total of 336 analyses. Each record is additionally scaled by the record-specific scaling factor for normalizing peak ground velocity, per FEMA P695. In addition, 600 synthetic ground motions were randomly generated using the Kanai-Tajimi spectrum [58,59], for a total of 936 time history analyses. For this demonstration, all ground motions are applied parallel to the exposed moment frame in Figure 7. The proposed methodology is only applicable when the building does not collapse, therefore only non-collapse analyses are considered. For a given analysis, collapse is determined by either global instability as indicated by a convergence failure in the analysis, or if any column plastic hinge rotation exceeds 0.06 radians. The value of 0.06 radians was chosen based on the maximum plastic rotation values from the ASCE 41 backbone curves used in the analysis; beyond this value a non-simulated flexure-shear or shear-induced axial failure is assumed to occur [57,60]. Of the 936 ground motions, 467 did not cause collapse. Any exterior beam or column that exhibited post-elastic behavior in any non-collapse case is considered a damage-sensitive component for the digital twin system. The identified beams and columns are highlighted in Figure 8.

### 3.3. BIM Development, UAV Survey and 3D Reconstruction

**Figure 9:**
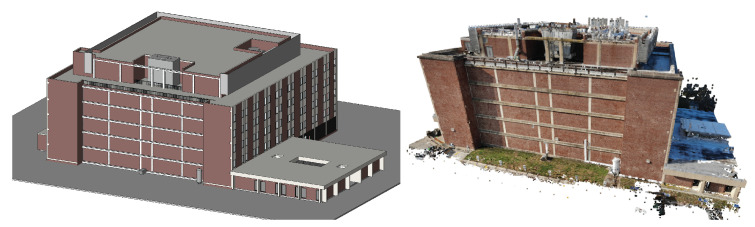
3D models of Turner Hall created for the digital twin. (**Left**): 3D BIM geometry. (**Right**): Point cloud generated from UAV images.

A BIM of Turner Hall containing the identified damage-sensitive components is developed starting from a preexisting model provided by the University of Illinois Facilities Department. The BIM geometry is shown in Figure 9. In the BIM, the exterior moment frame beams and columns are modeled, as well as several large mechanical components on the roof and ground levels that are identified as damage-sensitive components in the initial assessment.

A UAV survey was conducted over two days on 23 November and 2 December 2020, using a DJI Mavic Air drone with photographs taken every 1 s. On the first day, images were collected at 4056 × 2280 pixel resolution and on the second day, images were collected at 4056 × 3040 pixel resolution. A total of 989 images were collected, with a total size of 5.47 GB. Reality Capture [61], a commercial software package, is used to align and generate a 3D point cloud from the drone images. The maximum reprojection error for the registration is 2 pixels, with an average error of 0.71 pixels. The output point cloud is approximately 75 million points. Reality Capture outputs the intrinsic and extrinsic camera parameters as well as lens distortion parameters. A screenshot of the point cloud is shown in Figure 9.

Registration with the BIM is performed in Python and CloudCompare [62]. First, the point cloud from Reality Capture is imported to Python using Open3D [48], and downsampled in a uniform voxel grid to 5 million points. For registering the BIM and point cloud, a high fidelity point cloud is unnecessary and working with a downsampled cloud is more computationally efficient. Next, the BIM geometry is converted to a triangle mesh using IfcOpenShell [47] in Python. The BIM and point cloud are imported to CloudCompare and corresponding points are manually selected on each 3D model. Fifteen point correspondences are selected for the alignment. CloudCompare estimates a transformation matrix between the two models in a least squares sense. With this transformation, the root mean square error of the distances between corresponding points is 7.7 cm. Each UAV survey image is loaded into Python and undistorted using OpenCV [52], based on the lens parameters calculated by Reality Capture. The images’ camera parameters are imported and transformed to the BIM reference frame, based on the relations defined in Section 2. The digital twin can now be used for BIM-guided image selection and component identification. At this stage, the collection of registered UAV photographs, registered 3D point cloud, BIM, and structural analysis model form the digital twin.

### 3.4. Results and Discussion

This section demonstrates the application of BIM-guided component-wise image selection and BIM-guided component identification for the digital twin of Turner Hall, as described in Section 2. First, the BIM is used to sort through the UAV images and select an optimal set of images containing Turner Hall’s damage-sensitive components. Next, the UAV images are input to GrabCut for component identification. Sample results are presented in the following sections.

#### 3.4.1. BIM-Guided Image Selection by Component

**Figure 10:**
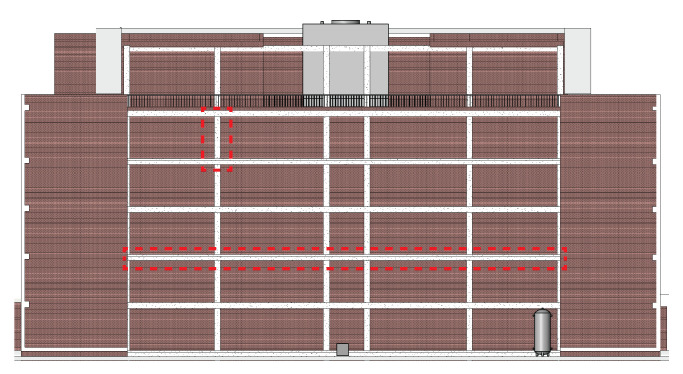
Example elements for automatic image selection are indicated by the dashed boxes.

Two exterior structural components are chosen to demonstrate automatic component-wise image selection. The first is a column between the fifth floor and roof; the second is a third-floor beam that spans the length of the building. Both elements are highlighted in Figure 10.

**Figure 11:**
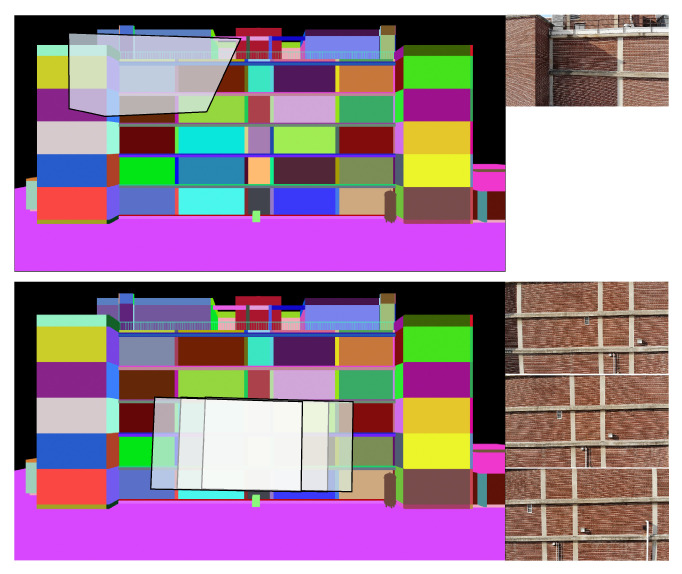
Automatic image selection results. The extents of the optimal images are projected to the BIM elevation. The original UAV images are displayed on the right.

A database mapping a list of BIM components to UAV images containing those components is generated using the procedure described in Section 2. Using this database, an optimal set of photos for each considered building component is selected from the set of nearly 1000 UAV images. For an actual inspection scenario, multiple UAV passes with the UAV camera tilted at different angles and at varying distances from the building will be necessary to obtain sufficient coverage and resolution [63], resulting in many times the number of images collected in this example. For the fifth-floor column, a single image is sufficient to give full coverage. The projected extent of the optimal image is shown in Figure 11. For the beam, the extents of the first three images, giving 72% coverage, are shown in Figure 11. Seven images are required to provide full coverage of the beam.

#### 3.4.2. BIM-Guided Component Identification

**Figure 12:**
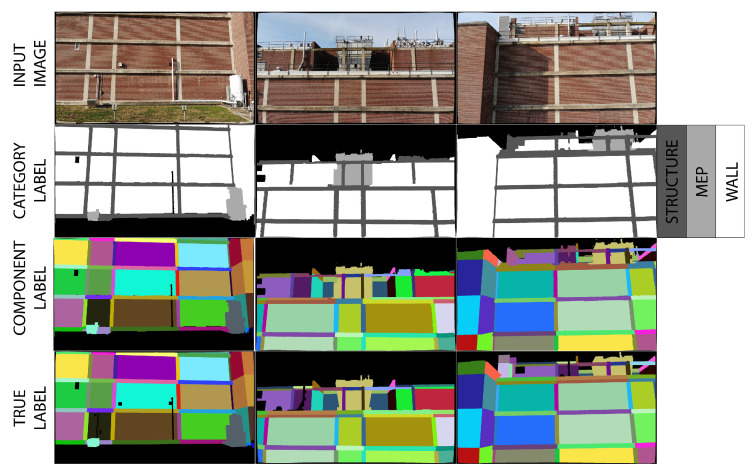
BIM-guided component identification results.

Three sample images are selected from the Turner Hall UAV survey to demonstrate automatic component identification. First, the BIM is rendered from the perspective of each undistorted input image, and initial foreground maps for each category (structure, wall, and MEP) are created. Initially, each pixel is assigned a category label, based on the foreground labels output by GrabCut for each of the three categories, as shown in Figure 12. Based on the initial category labels, each pixel is subsequently assigned a component label, and colored based on that component’s unique RGB identifier. Because this method is based on a nearest neighbor assignment, a maximum distance threshold of 50 pixels is set, beyond which pixels are assigned as background (black). Figure 12 shows the final component label and the true component label maps for comparison.

**Figure 13:**
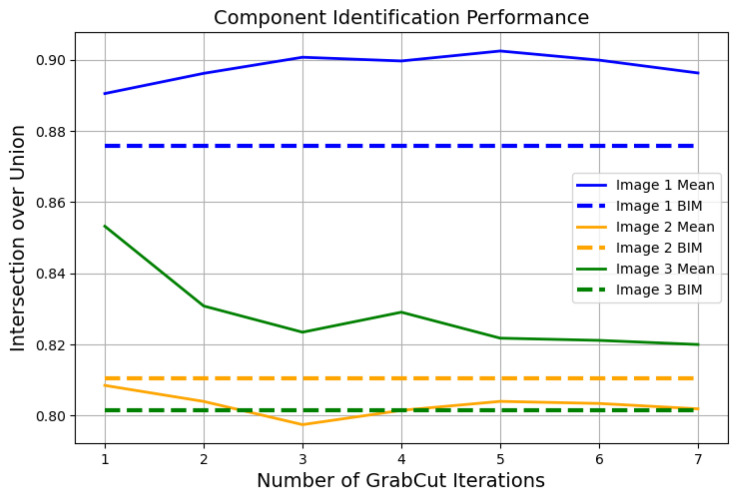
Average component identification performance as a function of number of GrabCut iterations performed (solid lines) compared to the performance of the unmodified BIM overlay (dashed lines).

The accuracy of the predicted component labels is assessed based on the manually assigned true labels in Figure 12. For each image, the algorithm is run five times for a set number of GrabCut iterations, ranging from 1 to 7 iterations. For comparison, the accuracy of the BIM rendered from the image perspective is also calculated. The intersection over union, IoU, where IoU = (number of true positives)/(true positives + false negatives + false positives), is used as a performance metric. The mean IoU of the five runs for each number of GrabCut iterations is presented, along with the IoU for the BIM, in Figure 13. Performance varies, with IoU for the automatically generated component labels and the BIM ranging from 0.80 to 0.90. For two of the three test images, the GrabCut-based method outperforms the BIM overlay. Additionally, the GrabCut algorithm has an advantage over the BIM overlay because it distinguishes small features not modeled in the BIM. For example, the GrabCut-based method segments MEP components like vents, lights, and pipes that do not appear in the BIM because they are not modeled. This advantage allows for a less detailed BIM to be included in the digital twin framework without compromising accuracy.

The workflow described in this section is intended primarily to allow structural damage identified in the images from a UAV survey to be linked to the associated component in the BIM. For example, exposed rebar and concrete spalling can be associated with a specific concrete moment frame column. Within the digital twin framework, each component will have a known relationship between observable damage and maximum earthquake demands, enabling assessment of the post-earthquake safety of the building system. The next example demonstrates an application of the digital twin for change detection between pre- and post-earthquake 3D point clouds for detecting large, volumetric changes due to nonstructural component damage, and associating this damage with specific nonstructural building components.

## 4. Example 2: 3D Change Detection for a Synthetic Earthquake-Damaged Masonry Veneer Wall

This section demonstrates use of the proposed framework for post-earthquake damage assessment of buildings using 3D change detection. However, this method requires images of both the baseline and post-earthquake building, which are not generally available. Rather, a synthetic graphics environment is created in which earthquake damage can be simulated. The building contained therein is similar in construction to Turner Hall; damage is imposed heuristically on a nonstructural masonry wall, based on reports of masonry damage described in [64]. In the remainder of this section, a description of the graphics model is presented, followed by a description of the point cloud generation and pre-processing steps. Finally, the results of the change detection are presented, with particular emphasis on how the BIM can be used to aggregate point cloud distance measurements to assess the damage states on individual building components.

### 4.1. Graphics Model Description

**Figure 14:**
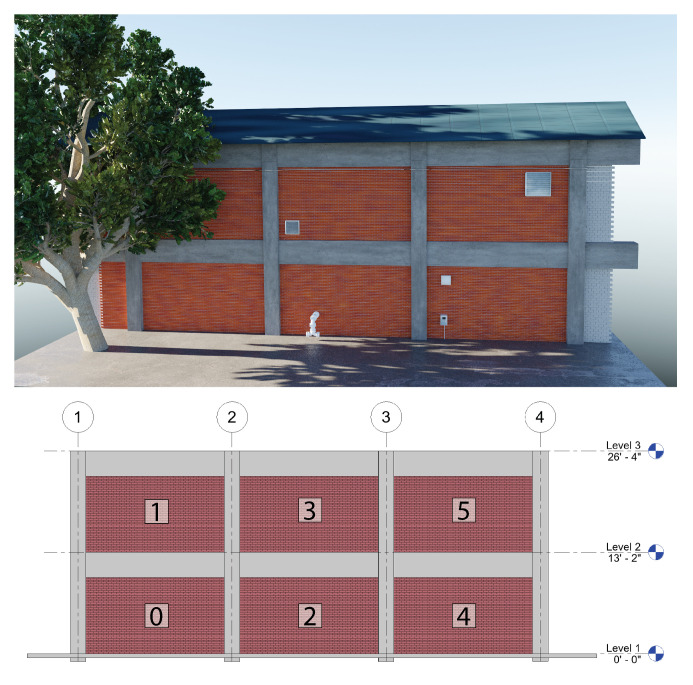
Synthetic graphics model created in Blender (**top**) and corresponding BIM created for element-wise damage assessment (**bottom**). Wall panel labels are indicated on the BIM.

The graphics model for this example, shown in Figure 14, is created in Blender [65], an open-source 3D modeling and rendering software. Materials and construction are modeled to mimic the design of Turner Hall. The concrete moment frame devides the facade into discrete masonry veneer wall panels. The masonry veneer is backed by a concrete masonry unit (CMU) wall. Additional detail elements, such as the tree, vents, and pipes, were downloaded from an online repository of 3D models [66]. The lighting direction and atmospheric conditions can be varied to reflect natural variations in the environmental conditions. A corresponding BIM is created using Revit (Figure 14) to enable BIM-guided component assessment. After creating the intact graphics model of the building, damage can then be applied at varying levels of severity.

**Figure 15:**
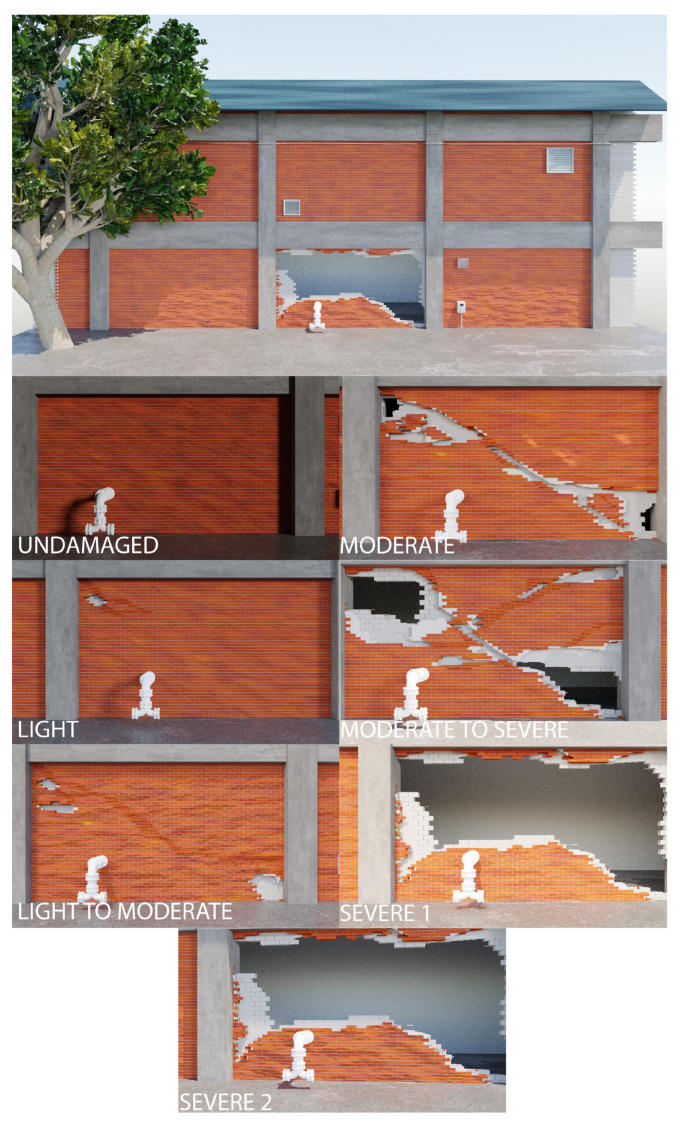
Different damage states in the graphics model from simulated UAV flights. Note that the perspective shifts in each damage state due to perturbations to the UAV flight path and lighting conditions.

Damage is applied to the model assuming in-plane shear failure of a single wall panel. The damage progression is based on photographs and descriptions of unreinforced masonry failures [64,67,68]. Initial damage is applied by removing mortar in a diagonal pattern from the top left to the bottom right of the wall panel to simulate shear cracking. As the damage level increases, clay bricks are removed to simulate partial collapse. Finally, in the most severe cases, the CMU blocks behind the clay masonry are removed to simulate full collapse of the wall. Figure 15 shows a rendering of the undamaged wall and of the five different damage levels considered: light, light to moderate, moderate, moderate to severe, and severe. In Figure 15, labels “Severe 1” and “Severe 2” refer to the same damaged wall geometry with different illumination and atmospheric conditions. A single wall panel is damaged while the rest are left intact to test robustness against varying illumination and camera angle for the undamaged panels.

A UAV flight is simulated in the graphics model for each damage level. As Figure 15 shows, for each level, the lighting and atmospheric conditions are randomly changed. A camera path is defined within the model, and as the camera moves along the defined path, it periodically renders the scene, producing a set of images analogous to a UAV survey of a building. The drone path is shifted slightly for each damaged model to simulate the variation in the drone path expected during multiple surveys of a physical building. This shift is illustrated in Figure 15, where each rendered image of the damaged wall is offset from every other image.

### 4.2. Point Cloud Generation and Pre-Processing

Point clouds are generated for each damage level from the simulated UAV flights. The simulated UAV images are input to Reality Capture [61] for 3D reconstruction to generate a point cloud for each damage state. For each of the damage levels, between 121 and 123 images are input to the 3D reconstruction; for the undamaged case, 159 images are used.

**Figure 16:**
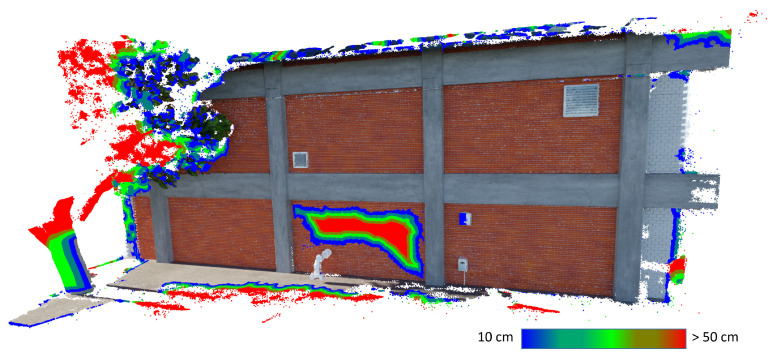
Distance measurements over 10 cm at each point in the source point cloud. Each point is assigned a color based on the measured distance to the target point cloud.

Several preprocessing steps are performed before change detection. The damaged and intact point clouds are manually aligned to the BIM by selecting point correspondences. The aligned clouds are uniformly downsampled to a 1 cm voxel grid, then input to Open3D’s statistical outlier removal function [48]. To compute the distance measurements between the two clouds, one cloud is designated as the source cloud and one is designated as the target cloud. Distances are measured from points in the source cloud to the points in the target cloud; the set of calculated distance measurements are associated with points in the source cloud. After preprocessing, the distances between the source cloud and the target cloud are calculated. Following Jafari et al [69] and Ghahremani et al [53], the distance measurements are taken as a proxy for volumetric changes to the structure. This relationship is demonstrated in Figure 16, which shows the distance measurements between the intact point cloud and severe damage case. Distance measurements are highest in the region around the applied damage. Subsequently, several postprocessing steps are performed to interpret the distance measures in terms of building component damage.

While the points in the damaged wall display high-distance measures, so do the poorly reconstructed regions around the tree and the perimeter of the point cloud, where there are fine details or a limited number of source images. To remove the influence of these spurious high-distance points, the BIM is used to assess each wall panel individually. Iterating through the six wall panels, the point cloud is cropped to the extents of the bounding box of the corresponding BIM element. To account for any misalignment with the BIM, the bounding box is scaled by a factor of 1.05 in the plane of the wall panel and by a factor of 2.5 perpendicular to the wall panel. For each point in the cropped source cloud, a local point density is calculated, which is defined based on the volume of the sphere containing the nearest 100 neighboring points. If the local point density is too low, then the regions around these points are considered to be poorly reconstructed and neglected. In this example, points are neglected if the log local point density is lower than the average log local point density. Statistics of the distance measures on the remaining points are then considered to determine the state of each individual wall panel. Mean and standard deviation are investigated as damage indicators. The methodology is applied twice: first, with the damaged point cloud as the source cloud and intact cloud as the target cloud, then with the intact cloud as the source cloud and damaged cloud as the target cloud.

### 4.3. Results and Discussion

**Figure 17:**
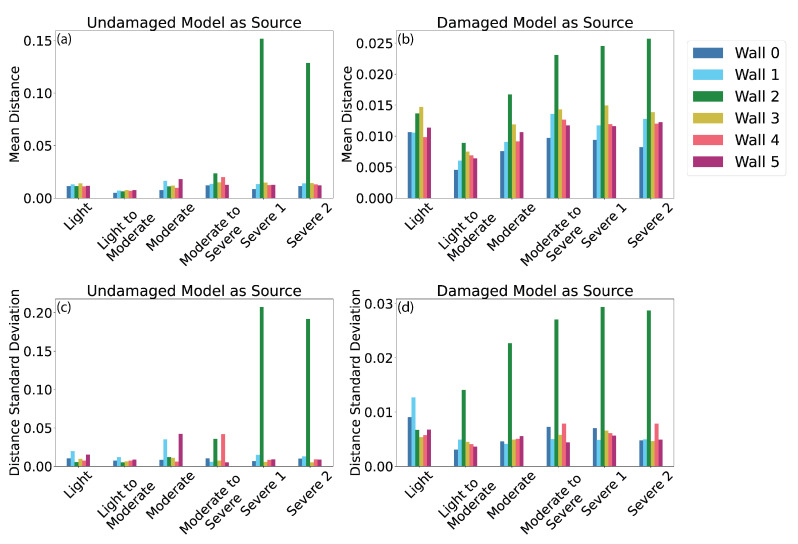
Results of 3D change detection. The left plots (**a**,**c**) use the intact model as the source cloud; the right plots (**b**,**d**) use the damaged model as the source cloud. The top plots (**a**,**b**) show the mean distance measure and the bottom plots (**c**,**d**) show the standard deviation of the distance measure for each wall panel at each damage level.

Figure 17 shows the results of the change detection methodology applied to the synthetic graphics model of the damaged masonry wall. In all four plots, Wall 2 is the damaged panel, and all other panels are undamaged. Refer to Figure 14 for the wall labels. Figure 17a,c show results with the intact cloud designated as the source cloud and Figure 17b,d show results with the damaged cloud as the source cloud. Figure 17a,b show the mean distance and Figure 17c,d show the standard deviation of distance for each wall panel for each damage level. Each cluster of bars in Figure 17 corresponds to a discrete damage level.

The best results are achieved when the damaged point cloud is assigned as the source cloud and standard deviation is used as the damage indicator. In Figure 17d, Wall 2 has a higher standard deviation of distance measures than the undamaged walls for all cases except light damage. Setting a threshold for damage at 0.010 identifies a single false positive (Wall 1, light damage) and a single false negative (Wall 2, light damage). At higher damage levels, a threshold of 0.010 always correctly identifies the damaged wall. The mean distance, as shown in Figure 17b, does not display as strong a difference between the damaged and intact wall panels. For the cases with the intact cloud as the source cloud (Figure 17a,c), both the mean and standard deviation reflect the damage to Wall 2 for the severe cases, but the method fails to distinguish the intact and damaged walls at lower levels of severity. Because the distance measurements are considered on a component-wise basis, no additional steps are needed to tie this damage to a specific wall panel.

In this second example, 3D point cloud change detection identifies damaged walls and automatically associates the damage with a specific BIM component. Because damage identification relies on a setting an appropriate threshold value, the method is best suited for components with binary damage states whose failure is associated with large displacements relative to the building, such as the collapsed wall in this example, or an overturned mechanical unit. As in the first example, the goal is to estimate a qualitative description of damage for assigning a fragility-consistent damage state. This assignment, in turn, will enable the observed damage to be linked with a structural analysis model for considering the safety of the full building.

The previous two examples demonstrated applications of individual elements of the digital twin system, but prior to future implementation, the full digital twin methodology must be demonstrated on an example building. Future research efforts will consist of a full validation using a physics-based graphics model (PBGM), a modeling approach that links a finite element analysis to a synthetic graphics environment, such as that used in this study, to photorealistically simulate structural damage [70]. Previous studies have used PBGMs to simulate concrete [9] and steel [71] damage. Efforts will include development of a PBGM to realistically simulate structural damage on Turner Hall, a simulated pre- and post-earthquake survey, and 2D and 3D damage detection using the synthetic imagery. Fully automated inspection will require rigorous testing and demonstration; at present, the methods proposed in this paper enable a hybrid approach, automating data collection and organization for human inspectors to review and interpret.

## 5. Conclusions

This study presented a BIM-based digital twin framework for predicting building performance from UAV photographic surveys. To date, the majority of the computer vision-based post-earthquake inspection research has been limited to component-level assessment. The studies have focused on identifying specific types of damage; work that has automatically estimated earthquake demands based identified on damage has been limited to individual components, without the extension to overall building performance. To address these limitations, this paper developed a digital twin framework that links components identified in UAV images or 3D point clouds to their corresponding component in a BIM. This link will enable component damage to be considered in terms of that component’s design, expected earthquake behavior, and relative importance to the safety and stability of the building system.

Two examples were presented to demonstrate the efficacy of the proposed approach. The first example demonstrated the development of the digital twin and its application for 2D images. The BIM guided relevant image selection, and then was applied, in combination with a GrabCut-based segmentation method, to assign each image pixel to an individual component. The second example demonstrated BIM-guided 3D change detection on point clouds, using a synthetic graphics environment. The 3D change identified moderately to heavily damaged nonstructural walls and is most effective at identifying nonstructural changes associated with large displacements relative to the main building. While these two examples demonstrated the potential of the proposed digital twin for assigning qualitative damage descriptions to building components. Nevertheless, the framework offers several opportunities for improvement.

During this study, some issues were identified for consideration in future research efforts. In the first example, the BIM is not a perfect reflection of the true building geometry; some components have simplified geometry, and not all building components are modeled. Moreover, the quality of the initial BIM alignment also affects the component labels, particularly at regions where high damage is anticipated, such as beam-column connections. These limitations demonstrate the need to accurately measure and document building components in the initial walkthrough so that the BIM geometry can better approximate the true geometry. In the second example, 3D change detection failed to identify light damage. The light damage case, corresponding to situations where the wall cracks but bricks do not crush or topple over, lacks large volumetric changes. In these cases, crack detection methods for 2D images would likely be more successful. Future research efforts will incorporate these proposed improvements for assessing both structural and nonstructural components in the digital twin.

Future research efforts will integrate a structural analysis into the digital twin system to achieve the goal of predicting global structural safety from a UAV survey. The structural analysis will enable component damage to be evaluated in the context of the component’s relative contribution to a damaged building’s remaining strength and stiffness to resist future earthquakes. Such a method will depend on the ability to accurately localize damage to specific components within the building, as presented in this study.
